# Supplementary material for: Raman Spectroscopic Analysis to Detect Reduced Bone Quality after Sciatic Neurectomy in Mice
Source: Molecules. 2018 Nov 25;23(12):3081. doi: 10.3390/molecules23123081 (PMC6321365; doi:10.3390/molecules23123081)
Supplement: Supplementary file 1 [file molecules-23-03081-s001.pdf]

Table S1. Summary of bone quality assessments based on vibrational spectroscopy.

		Human fracture[21]			Human aging[40]			Rat aging[22]			Mice growing[23]			Rat OVX[24]		
		ratio	N	p value	ratio	N	p value	ratio	N	p value	ratio	N	p value	ratio	N	p value
Raman spectroscopy/FT-IR		Fracture/Non fractured			Elder (85y)/Younger (52y)			3 month/24 month			Postnatal day1/6 month			OVX/Sham		
Carbonate/phosphate		1.012	15/11	0.51	1.050	16 (Total)	<0.05	1.231	5/4	<0.01	1.520	2/2	-	1.094	8/8	<0.05
mineral/matrix	Mineral/phenylalanine															
	Mineral/proline+hydroxyproline															
	Mineral/CH_2				1.073	16 (Total)	<0.05									
	Mineral/Amide I	1.322	15/11	0.11				1.133	5/4	<0.01	1.838	2/2	-	0.900	8/8	<0.05
Amide I/CH_2																

		Rat SCI[25]			Rat CKD[41]			Rat DM[42]			Rat EX[43]			Mice NX (our result)		
		ratio	N	p value	ratio	N	p value	ratio	N	p value	ratio	N	p value	ratio	N	p value
Raman spectroscopy/FT-IR		SCI/Sham			CKD/Control			DM/Control			Exercise/Control			NX/Sham		
Carbonate/phosphate		1.077	6/6	<0.05	1.250	6/6	<0.05	1.02	14/18	0.92	0.858	15/15	<0.022	1.048	7/3	<0.01
mineral/matrix	Mineral/phenylalanine													1.128	7/3	<0.01
	Mineral/proline+hydroxyproline										1.152	15/15	<0.027	1.056	7/3	<0.01
	Mineral/CH_2	0.714	6/6	<0.05										1.036	7/3	0.14
	Mineral/Amide I				1.250	6/6	<0.05	1.17	14/18	<0.01				1.061	7/3	0.20
Amide I/CH_2														0.956	7/3	0.05
